# Characteristics of hospitalization patterns and expenditures in cross-border medical tourism: a knee replacement surgery cohort study

**DOI:** 10.3389/fpubh.2025.1655280

**Published:** 2025-12-11

**Authors:** Shiya Zuo, Ling Lin

**Affiliations:** 1 Department of Pediatric Dentistry, School and Hospital of Stomatology, Guangdong Engineering Research Center of Oral Restoration and Reconstruction & Guangzhou Key Laboratory of Basic and Applied Research of Oral Regenerative Medicine, Guangzhou Medical University, Guangzhou, Guangdong, China; 2 The University of Hong Kong-Shenzhen Hospital, Shenzhen, China

**Keywords:** seeking medical care in mainland China, cross-border medical cooperation, knee arthroplasty, length of stay, hospitalization costs

## Abstract

**Objective:**

Using knee arthroplasty as a case study, this research explores the characteristics of length of stay (LOS) and hospitalization costs for Hong Kong residents receiving medical treatment in mainland China.

**Methods:**

Utilizing front-page medical record data of patients who underwent knee arthroplasty at Hospital H, descriptive statistics, univariate analysis, and mediation effect tests were conducted to analyze the impact of being a Hong Kong patient on LOS and hospitalization costs.

**Results:**

The study included 356 patients, predominantly older adults over 65 years old (77.25%), with a similar gender distribution. Hong Kong patients had shorter LOS, shorter postoperative LOS, and lower hospitalization costs, laboratory and examination costs, and medication costs. LOS fully mediated the effect of being a Hong Kong patient on hospitalization costs.

**Conclusion:**

Hong Kong residents seeking medical care in mainland China are driven by factors distinct from those of non-local patients within mainland China, with medical quality, efficiency, and cost being significant drivers of cross-border healthcare seeking. It is essential to strengthen cross-border medical collaboration between Shenzhen and Hong Kong, and the Greater Bay Area as a whole, through institutional mechanisms, welfare benefits, long-term follow-up, and health monitoring to ensure tangible medical benefits for Hong Kong patients.

## 1 Introduction

The construction of healthcare integration in the Guangdong-Hong Kong-Macao Greater Bay Area has provided institutional breakthroughs for innovations in cross-border medical services. Since the *Outline Development Plan for the Guangdong-Hong Kong-Macao Greater Bay Area* proposed the “Healthy Bay Area” strategy, policymakers have gradually established a multidimensional framework for Shenzhen-Hong Kong medical collaboration. This framework includes opening cross-border practice qualifications for Hong Kong doctors, optimizing access mechanisms for pharmaceuticals and medical devices from Hong Kong and Macao, and promoting mutual recognition of hospital accreditation standards between the two regions (1, 2). These measures systematically address institutional barriers to cross-border healthcare resource flows. This policy system not only facilitates northbound healthcare-seeking for Hong Kong residents but also reshapes the spatial distribution of medical resource allocation within the Greater Bay Area through deep integration of healthcare service supply (1).

Under the impact of special public health events, the healthcare system in Hong Kong has faced practical pressures such as a backlog of elective surgeries and extended service delivery cycles, which further intensified the “demand spillover” effect of cross-border medical care (3). During this period, medical institutions in mainland China, leveraging their efficient service response capabilities, became a significant supplementary force in alleviating the healthcare difficulties faced by Hong Kong residents. Existing research on cross-border medical behaviors has primarily focused on medical tourism, border medical care, and healthcare demands arising from cross-border work. The driving forces behind patient mobility include shorter distances, higher-quality medical services, or lower medical costs, exemplified by individuals traveling to Hungary for dental and orthopedic treatments or UK-supported NHS patients seeking care in Brussels and France in the early 2000s. Notably, previous studies have generally identified a “dual-high” phenomenon—longer length of stay (LOS) and higher medical costs—among non-local patient groups compared to local patients at the destination (4, 5). The reasons for this involve both objective differences in patient case complexity and the relative highlighting effect, under payment policy reforms, of better cost control for local patients compared to non-local patients (4).

When Hong Kong residents seek medical treatment in mainland China, do their characteristics of length of stay and costs still align with the patterns observed in non-local medical care models? On the one hand, compared to domestic non-local medical care within China, which often occurs within the same social system, medical seeking from Hong Kong to the mainland involves crossing different social systems. Does this confer new characteristics in terms of hospitalization costs and LOS? On the other hand, compared to existing international cross-border medical care, which often occurs across nations but within similar social systems, this scenario is distinct. Therefore, this study focuses on analyzing the characteristics of LOS and hospitalization costs for voluntary cross-border medical care within a single country but across different social systems.

This study focuses on knee arthroplasty procedures performed in the orthopedic department of Hospital H, a preferred institution for Hong Kong patients. As a demonstrative platform for Shenzhen-Hong Kong medical cooperation, Hospital H recorded 3,676 inpatient discharges and 128,500 outpatient and emergency visits for Hong Kong patients in 2023. The orthopedic department had the highest number of inpatient discharges among Hong Kong patients, with knee arthroplasty (including bicompartmental and total knee arthroplasty) being the most common procedure. Hospital H has established a significant brand aggregation effect in the field of joint arthroplasty. Its patient volume and procedural standardization level provide an ideal observation window for studying cross-border medical care. Furthermore, hospital H operates under a fixed annual salary system modeled after Hong Kong's public hospital compensation structure. This system eliminates any financial incentive for physicians to treat local and cross-border patients differently. Building upon the homogeneous clinical pathway for knee arthroplasty, this unique salary model at Hospital H, and the concentrated flow of cross-border patients, this study aims to descriptively analyze the differences in length of stay, costs, and related patterns between Hong Kong medical tourists and local mainland patients undergoing elective arthroplasty at a specific center in mainland China. It further seeks to explore the underlying reasons and policy implications.

## 2 Materials and methods

### 2.1 Sample and data sources

Case data for this study were extracted from the front pages of medical records of patients who underwent knee arthroplasty at Hospital H between July 2023 and June 2024, totaling 363 cases. To ensure data validity and analytical accuracy, data cleaning was performed to verify consistency between ICD-9-CM3 procedure codes and Chinese surgical names, with the primary procedures identified as bicompartmental knee arthroplasty or total knee arthroplasty. Patients with missing addresses, bilateral knee arthroplasty, hospitalization costs exceeding three times the mean were excluded. The final study cohort comprised 356 patients (see Supplementary Figure 1). Their demographic characteristics showed no statistically significant differences from the original sample upon statistical analysis and comparison, as detailed in Supplementary Table 1.

### 2.2 Variable definitions

The independent variable in this study was “whether the patient is a resident of Hong Kong” (identified based on permanent residence being Hong Kong). The dependent variables were “length of hospital stay” and “hospitalization costs,” with “length of hospital stay” also serving as the mediating variable. Existing research suggests that hospitalization costs and length of stay for knee arthroplasty are primarily influenced by disease-related factors such as comorbidities, cost of medical consumables, history of prior knee arthroplasty, and anesthesia method, as well as demographic factors like gender and age. Socioeconomic factors, including family support and personal financial conditions, also play a role (6–8). Considering data availability, this study ultimately incorporated the following measurement indicators: age and gender for demographic information, marital status for family support, comorbidity status (Carlson Comorbidity Index, CCI score) for comorbidities, and anesthesia method (spinal anesthesia/general anesthesia) and history of prior knee arthroplasty for disease and treatment characteristics. The CCI score was calculated by extracting ICD-10 codes from other diagnoses listed on the front page of medical records and computing a total score based on the CCI index calculation table. Total hospitalization costs include only the expenses incurred during the hospital stay and do not cover post-discharge care costs.

### 2.3 Research hypotheses

H1: Hong Kong patients have a longer length of hospital stay compared to mainland patients.

H2: Hong Kong patients incur higher hospitalization costs compared to mainland patients.

### 2.4 Statistical analysis methods

Data organization and analysis were performed using SPSS 25.0 statistical software. Mediation effect testing was conducted using the Process v2.13 plugin. Statistical analysis methods included descriptive statistics, univariate analysis, and mediation effect testing (Bootstrap method).

## 3 Results

### 3.1 Demographic and clinical characteristics

As shown in Table 1, the study included 356 patients undergoing unilateral knee arthroplasty, with 213 (59.83%) being Hong Kong residents. The majority (217, 60.96%) were aged 65–75 years, with a higher proportion of females (206, 57.87%) than males. Most patients (278, 78.09%) were married and not divorced. Clinically, most patients (205, 57.58%) had a CCI score of 0, 221 (62.08%) underwent spinal anesthesia, and 68 (19.10%) had a prior history of knee arthroplasty.

**Table 1:** Demographic and clinical characteristics of the sample.

**Variables**	**Category**	**Mainland patients 143 (40.17%)**	**Hong Kong patients 213 (59.83%)**	**Total 356 (100%)**	**t/^2^**	***P***
Age	y ≤ 65	31 (21.68%)	50 (23.47%)	81 (22.75%)	0.396	0.82
	65 < y ≤ 75	90 (62.94%)	127 (59.62%)	217 (60.96%)		
	y>75	22 (15.38%)	36 (16.90%)	58 (16.29%)		
Sex	Male	42 (29.37%)	108 (50.70%)	150 (42.13%)	15.971	< 0.001^***^
	Female	101 (70.63%)	105 (49.30%)	206 (57.87%)		
Marital status	Currently single	23 (16.08%)	55 (25.82%)	78 (21.91%)	4.742	0.029^*^
	Married with partner	120 (83.92%)	158 (74.18%)	278 (78.09%)		
CCI score	0	80 (55.94%)	125 (58.69%)	205 (57.58%)	0.556	0.754
	1	47 (32.87%)	69 (32.39%)	116 (32.58%)		
	≥2	16 (11.19%)	19 (8.92%)	35 (9.83%)		
Anesthesia type	Spinal anesthesia	84 (58.74%)	137 (64.32%)	221 (62.08%)	1.131	0.288
	General anesthesia	59 (41.26%)	76 (35.68%)	135 (37.92%)		
Prior knee arthroplasty	No	117 (81.82%)	171 (80.28%)	288 (80.90%)	0.131	0.718
	Yes	26 (18.18%)	42 (19.72%)	68 (19.10%)		
Hospital stay duration		9.87 ± 4.07	8.93 ± 3.02	9.31 ± 3.50	6.156	0.014^*^
Preoperative days		3.19 ± 1.95	3.04 ± 1.75	3.10 ± 1.83	0.546	0.460
Postoperative days		6.68 ± 3.40	5.89 ± 2.55	6.21 ± 2.94	6.193	0.013^*^
Medical service fees		10,728.91 ± 2,333.31	10,282.07 ± 1,783.42	10,461.56 ± 2,031.07	4.178	0.042^*^
Examination/laboratory fees		2,655.57 ± 804.84	2,459.11 ± 811.84	2,538.03 ± 813.63	5.045	0.025^*^
Material costs		12,799.82 ± 1,235.31	12,856.64 ± 1,095.11	12,833.82 ± 1,152.11	0.208	0.649
Medication costs		803.99 ± 547.15	670.36 ± 296.16	724.04 ± 420.04	8.852	0.003^**^
Hospital-acquired infections		0	0	0	–	–
Total costs		26,988.29 ± 3,285.29	26,268.18 ± 2,737.06	26,557.44 ± 2,985.97	5.033	0.025^*^

^*^P < 0.05; ^**^P < 0.01; ^***^P < 0.001.

Age and CCI index were adjusted using the Bonferroni correction.

### 3.2 Univariate analysis

As presented in Table 1, compared to non-Hong Kong patients, Hong Kong patients showed a higher proportion of females, a lower proportion of married individuals, shorter hospital stay durations, and lower hospitalization costs. No significant differences were observed in prior knee arthroplasty history, anesthesia type, or comorbidity status. The shorter hospital stay duration was attributed to reduced postoperative hospitalization time, with no significant difference in preoperative stay. Regarding costs, Hong Kong patients incurred lower medical service fees, examination/laboratory fees, and medication costs, except for material costs. Regarding medical quality, none of the knee arthroplasty patients experienced complications or hospital-acquired infections during their hospitalization.

### 3.3 Mediation effect test of length of hospital stay

A mediation effect test was conducted with hospitalization costs as the dependent variable, length of hospital stay as the mediating variable, whether the patient was from Hong Kong as the independent variable, and age, gender, marital status, comorbidities, anesthesia method, and history of prior knee arthroplasty as control variables. The results are presented in Table 2. After controlling for other factors, Hong Kong patients undergoing knee arthroplasty had an average hospital stay 0.93 days shorter than non-Hong Kong patients. Compared to the group aged 65 and below, the average length of stay increased by 0.35 days and 1.72 days for the groups aged 65–75 and over 75, respectively. Patients who were married with a living spouse or not divorced had an average hospital stay 1.23 days shorter than those who were unmarried, divorced, or widowed. In contrast, gender, comorbidities, and history of prior knee arthroplasty had no significant impact on the length of stay.

**Table 2:** Results of multivariable linear regression analysis and mediation effect test for hospital stay duration and hospitalization costs in patients undergoing knee arthroplasty.

**Variable**	**Mediator: hospital stay duration**				**Dependent variable: hospitalization costs**			
	***Coeff***	***Boot Mean***	***LLCI***	***ULCI***	***Coeff***	***Boot Mean***	***LLCI***	***ULCI***
constant	8.92	8.95	6.87	11.00	19,323.05	19,323.97	18,182.00	20,416.07
HK patient	−0.93	−0.94	−1.70	−0.21	16.30	12.32	−363.80	399.79
Married with partner	−1.24	−1.24	−2.38	−0.18	−174.52	−179.67	−611.62	250.51
General anesthesia	0.50	0.49	−0.25	1.26	685.84	689.75	264.61	1,111.37
Prior knee arthroplasty	−0.48	−0.49	−1.35	0.41	−731.57	−729.09	−1,214.74	−280.27
Female	0.36	0.36	−0.34	1.04	61.05	60.85	−296.22	428.29
**Age (ref:** ≤ **65 years)**								
65 < y ≤ 75	0.35	0.35	−0.45	1.13	101.03	102.69	−345.41	533.69
y>75	1.72	1.71	0.51	2.91	437.12	433.95	−216.07	1,062.50
**CCI score (ref: 0)**								
1	0.53	0.53	−0.26	1.36	674.34	668.50	264.40	1,079.89
≥2	0.72	0.70	−0.51	2.00	1,105.19	1,095.93	446.24	1,821.56
Hospital stay duration	641.68	641.81	578.45	707.30
*R*^2^ = 0.822, *P* < 0.001					*R*^2^ = 0.397, *P* < 0.001			
	***Effect***	***BootSE***	***BootLLCI***	***BootULCI***				
X → Y	16.30	196.19	−369.59	402.19				
X → M → Y	−599.23	243.77	−1,097.71	−133.79				

The analysis excluded variables with VIF >2 to mitigate multicollinearity.

Bootstrap samples = 5,000, Confidence level for confidence intervals = 95%.

After controlling for other factors, there was no significant difference in average hospitalization costs between Hong Kong and non-Hong Kong patients undergoing knee arthroplasty. Compared to patients with a CCI score of 0, the average hospitalization costs increased by CNY 649.44 and CNY 1,082.54 for those with scores of 1 and 2 or above, respectively. Patients who received general anesthesia had an average hospitalization cost increase of CNY 731.74 compared to those who received spinal anesthesia. For each additional day of hospital stay, the average hospitalization cost increased by CNY 641.73. At this point, age, gender, marital status, and other factors had no significant impact on hospitalization costs.

The length of hospital stay fully mediated the effect of whether the patient was from Hong Kong on hospitalization costs.

### 3.4 Mediation effect analysis of hospitalization and cost structure

Using hospitalization costs as the dependent variable, and preoperative length of stay, postoperative length of stay, medical service fees, laboratory and examination fees, material costs, and medication costs as mediating variables, with Hong Kong patient status as the independent variable and age, gender, marital status, comorbidities, anesthesia method, and history of prior knee arthroplasty as control variables, a mediation effect analysis was conducted. The results, as shown in Supplementary Tables 1–6, are as follows: the mediation effect of preoperative length of stay was not significant; postoperative length of stay fully mediated the effect of Hong Kong patient status on hospitalization costs; medical service fees partially mediated this effect; laboratory and examination fees fully mediated the effect; the mediation effect of material costs was not significant; and medication costs fully mediated the effect of Hong Kong patient status on hospitalization costs. This indicates that Hong Kong patients had shorter postoperative hospital stays and lower laboratory/examination and medication costs, while no significant differences were observed in preoperative length of stay or material costs compared to mainland patients.

## 4 Discussion and conclusion

The results of this study indicate that Hong Kong patients undergoing knee arthroplasty exhibited both lower hospitalization costs and shorter lengths of stay, contrasting with the “dual-high” phenomenon observed in domestic non-local medical care. This suggests that the drivers for Hong Kong patients seeking medical care outside their region may differ from those in other parts of the country. Mediation effect analysis revealed that the length of hospital stay fully mediated the impact of Hong Kong patient status on hospitalization costs. Structurally, Hong Kong patients had shorter postoperative hospital stays and lower laboratory/examination and medication costs. However, in terms of healthcare quality, none of the patients acquired a hospital infection during their hospitalization, indicating that the quality of care was not compromised. Nevertheless, due to the lack of long-term follow-up data, we cannot assess whether the long-term outcomes are comparable. Thus, this study demonstrates that for knee arthroplasty, Hong Kong patients seeking medical care in mainland China can achieve a balance among medical efficiency, quality, and economic benefits.

### 4.1 Institutional drivers for optimized hospitalization cycles among Hong Kong patients

The findings of this study are completely opposite to the initial hypotheses (H1, H2). The study originally hypothesized that Hong Kong patients would have longer hospital stays and higher costs. This assumption was potentially based on factors faced by cross-border patients, such as language and cultural barriers, unfamiliarity with the local healthcare system, and potentially more complex preoperative conditions, all of which could prolong hospitalization and increase medical expenses (9, 10). However, this hypothesis overlooked a more critical determinant: the unique healthcare-seeking motivations of the medical tourist cohort.

On one hand, the primary motivation for Hong Kong patients seeking care in mainland China is likely to circumvent the protracted waiting times in Hong Kong's public hospitals. Although the Hong Kong public healthcare system adheres to the principle of universal access (2), it faces persistent pressure from long waiting lists for elective surgeries. As of June 30, 2024, the number of patients waiting for total joint replacement in Hong Kong reached 33,951, with the median waiting time being longest in Kowloon West at 51 months and shortest in New Territories West at 7 months (3). This situation objectively creates a push factor for patients to seek care in regions with higher service efficiency. On the other hand, evidence suggests that if the waiting time for knee arthroplasty exceeds three months, it can lead to increased healthcare costs and reduced health-related quality of life (HRQoL) over the subsequent decade (11). In a different context, another study indicates that Australia's knee arthroplasty registry represents a cost-effective undertaking for the government (12).

From the patient's perspective, when considering healthcare accessibility, safety, and economic factors, the inability of the local Hong Kong system to meet timely demand makes seeking cross-border alternatives a rational choice to overcome resource constraints. It is noteworthy that although the Hong Kong SAR government has attempted to alleviate cross-border healthcare barriers through extended welfare schemes, institutional gaps in inpatient coverage remain, leaving patients highly sensitive to indirect costs. Currently, no Hong Kong government medical welfare schemes provide coverage for inpatient care; arrangements like the Elderly Health Care Voucher are only applicable to outpatient services. Consequently, these patients prioritize highly efficient and expedited services. Once clinical discharge criteria are met, they have a strong incentive to be discharged quickly and return to Hong Kong.

On the other hand, Hospital H operates under a fixed salary system, eliminating any financial incentive for physicians to prolong hospital stays or increase services for revenue generation. For self-paying Hong Kong patients, shortening the LOS directly reduces their total costs. The standardized clinical pathway management implemented at Hospital H effectively balances healthcare quality with patient preferences. By employing flexible discharge mechanisms that return postoperative decision-making power to patients, this institutional design aligns supply-side capabilities with demand-side preferences, constituting a key factor in reducing the hospitalization cycle.

Therefore, the rejection of our research hypotheses powerfully demonstrates that under specific institutional arrangements and driven by patient motivations, cross-border healthcare can manifest as a model characterized by higher efficiency and lower costs. This finding provides a fresh perspective for understanding the diverse models of cross-border medical care. Furthermore, although the mediation analysis confirmed that LOS is a key mechanism explaining the cost differences—a relatively direct causal pathway—this study lacks qualitative data (e.g., in-depth patient decision interviews, healthcare provider perspectives) to uncover the root causes behind the observed differences in LOS. Investigating these underlying reasons represents a crucial direction for future research.

### 4.2 Cross-border synergistic effects in cost control mechanisms

This study found that the reduction in hospitalization costs was primarily associated with decreases in laboratory/examination fees and medication costs, while no significant change was observed in material costs. This may be related to the clinical pathway for knee arthroplasty, where postoperative expenses largely consist of imaging evaluations, laboratory monitoring for conditions such as thrombosis, and daily prophylactic medications for infection and thrombosis prevention (13). The shortened hospital stay may have directly influenced the arrangement of postoperative monitoring and medication use, or partially shifted inpatient medical services to outpatient care in Hong Kong (13, 14). However, due to the lack of available data, it is not possible to further investigate whether these medical measures were implemented during subsequent follow-ups.

The differentiated performance of cross-border medical costs reveals a deeper interaction between payment systems and organizational incentives. Unlike the conventional pattern in which non-local patients in mainland China incur higher costs due to treatment complexity, the cost control observed among Hong Kong patients stems from multi-system synergies: First, the decision-making motivation for cross-border medical care has shifted from “technical attraction” to “efficiency priority,” reducing the potential for cost premiums associated with critical conditions (5, 15). Second, the fixed salary system at Hospital H, modeled after Hong Kong's public sector, severs the link between physician income and medical practices, transforming clinical pathway implementation from passive compliance to active optimization (16). Third, the integration of healthcare management philosophies between Shenzhen and Hong Kong has fostered a unique cost containment mechanism—while hospitals in mainland China generally face pressure to shift costs under payment reform, Hospital H has achieved endogenous compression of hospitalization costs through institutional coupling (17, 18). This cross-border synergistic cost-control pathway suggests that the marginal benefits of payment policy adjustments may be limited and need to resonate with organizational reforms in personnel management and quality culture (19, 20).

### 4.3 Conclusion

This study demonstrates that the hospitalization duration and cost characteristics of Hong Kong residents seeking medical care in mainland China reflect a unique cross-border healthcare logic: the long-standing pressure from elective surgery waiting times within Hong Kong's public healthcare system, coupled with gaps in cross-border inpatient coverage, drives patients toward efficiency-oriented mainland institutions to achieve optimal outcomes in medical quality, efficiency, and cost at the individual level. By implementing standardized clinical pathways and flexible discharge mechanisms, Hospital H ensures medical quality while meeting patients' demands for autonomous decision-making during postoperative recovery, forming the core driver for optimized hospitalization cycles. At the cost control level, the institutional synergy between Shenzhen and Hong Kong is the fixed salary system eliminates financial incentives for overutilization of medical services, while efficiency-driven healthcare decisions reduce non-essential service consumption, collectively shaping a cost-containment paradigm for cross-border care. However, due to the lack of long-term follow-up data, it remains unclear whether accelerated discharge affects long-term outcomes or offsets the survival benefits gained from timely treatment. This study indicates that current Shenzhen-Hong Kong medical collaboration has evolved from mere service substitution to systemic rule restructuring, urgently requiring institutional innovations to overcome barriers to resource mobility and achieve a transition from short-term resource complementarity to long-term system integration.

### 4.4 Policy recommendations

First, establish a tiered cross-border healthcare security system. Priority should be given to expanding direct payment channels for inpatient costs incurred by Hong Kong residents in the mainland, and exploring the development of cross-border settlement platforms for commercial health insurance. This would gradually upgrade the current transitional “welfare portability” into institutionalized arrangements. Second, innovate cross-border tiered healthcare collaboration models. Leveraging Hong Kong's specialist expertise and the mainland's primary care network, joint efforts should be made to develop referral guidelines and teleconsultation mechanisms. This would facilitate the diversion of elective surgeries such as knee arthroplasty to the mainland, while preserving Hong Kong's core role in managing complex cases. Third, strengthen postoperative follow-up and the monitoring of patient-reported outcomes. By utilizing information technology, we can achieve remote high-quality follow-ups, health monitoring, and health education, thereby better evaluating the effectiveness of cross-border medical services.

### 4.5 Study limitations

(1) The most significant limitation of this study is selection bias. The Hong Kong patient cohort represents a self-selected group who may possess greater payment capacity, higher health literacy, and more urgent healthcare needs, while simultaneously facing the pressure of out-of-pocket payments. Consequently, the observed differences in length of stay and costs between them and local patients likely reflect these pre-existing characteristics rather than a pure effect of geographical origin. Therefore, the findings of this study should be interpreted as a description of a specific medical tourism model, not as conclusions from a strict comparative effectiveness analysis.

(2) The study's sample size is relatively limited, drawing data only from patients who underwent knee arthroplasty at Hospital H within a one-year period, which may be insufficient to fully represent the overall landscape of northbound healthcare-seeking from Hong Kong. Furthermore, the study focused solely on knee arthroplasty and did not encompass other types of cross-border medical services, thus the generalizability of the conclusions requires further validation. Although multiple influencing factors were considered, other unmeasured variables might also affect length of stay and costs, potentially limiting the precision of the analytical results. Moreover, the absence of long-term follow-up data prevents any assessment of long-term efficacy benefits.

(3) The hospitalization costs in this study only reflect the direct costs incurred during the hospital stay from the healthcare provider's perspective. They do not include the additional expenses borne by Hong Kong patients and their families, such as transportation, accommodation, meals, and potential costs for accompanying persons. These costs effectively shift a portion of the economic burden from the healthcare system to patient families. Similarly, post-discharge expenses for rehabilitation, follow-up visits, or complication management were not included. Thus, our conclusion of lower costs is strictly confined to the inpatient hospitalization period.

(4) The conclusions of this study are primarily applicable to medical institutions that have implemented a fixed annual salary system. This characteristic of Hospital H makes it not broadly representative of hospitals in mainland China, and caution is warranted when extrapolating these findings to settings where fixed salaries constitute a low proportion of physician compensation. However, as China is currently promoting an increase in the proportion of fixed salaries within physician compensation from the top down, this study may hold some reference value for future healthcare scenarios.

Future research should expand sample sizes, integrate patient-reported outcomes, link outpatient and inpatient data, broaden the range of diseases studied, and comprehensively calculate the full patient disease burden. This would enable a precise assessment of the short-, medium-, and long-term impacts of changes in costs and efficiency, thereby enhancing the reliability, applicability, and practical significance of the conclusions.

## Data Availability

The original contributions presented in the study are included in the article/Supplementary material, further inquiries can be directed to the corresponding author.
