# Supplementary material for: Characteristics of hospitalization patterns and expenditures in cross-border medical tourism: a knee replacement surgery cohort study
Source: Front Public Health. 2025 Dec 11;13:1655280. doi: 10.3389/fpubh.2025.1655280 (PMC12738367; doi:10.3389/fpubh.2025.1655280)
Supplement: Supplementary file 1 [file Data_Sheet_1.docx]

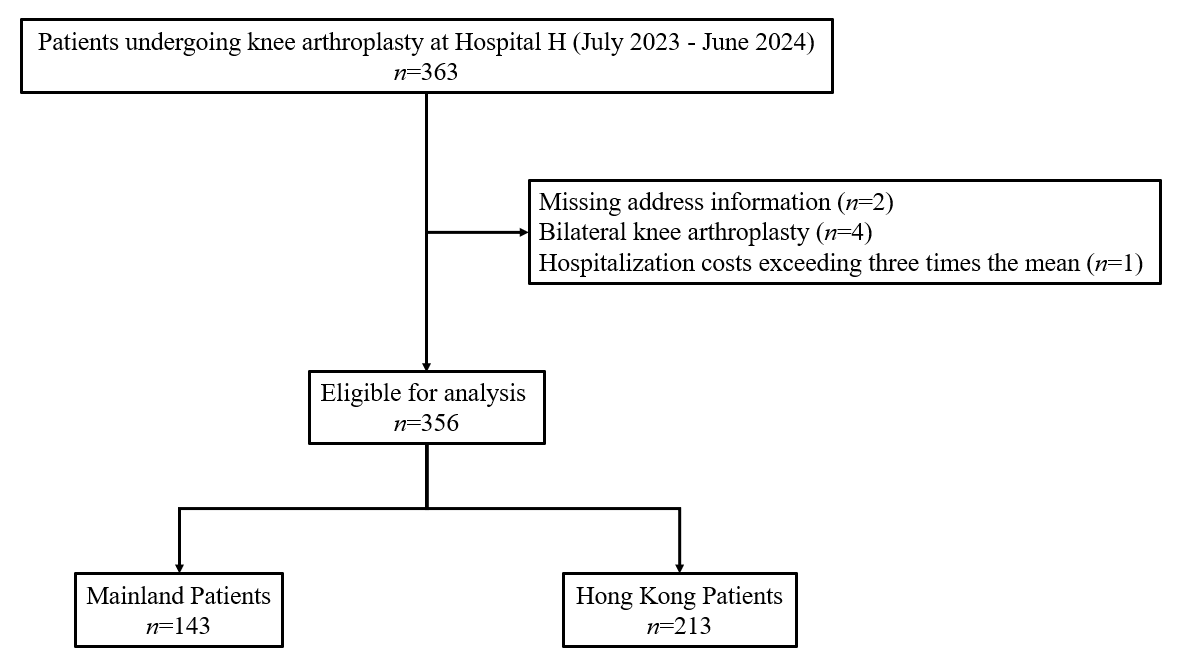


**Supplemental Figure 1**

*Note: The following cases were excluded from this study: patients with missing addresses (as this precluded group assignment), those who underwent bilateral knee arthroplasty (due to significantly higher material costs compared to unilateral procedures, making them incomparable), and one patient with hospitalization costs exceeding three times the mean. A review of the cost records for this outlier revealed the use of customized medical materials, with total material costs reaching 67,523.16 CNY—significantly higher than the mean of 12,833.82 CNY. Other cost components for this patient, including medical services (15,983.26 CNY), examinations and tests (6,430.94 CNY), and medications (899.10 CNY), were also slightly elevated compared to their respective means (10,461.56 CNY, 2,538.03 CNY, and 724.04 CNY). Given that this was the only case among the 363 patients involving high-cost customized materials—a patient-driven choice—it was excluded to maintain dataset consistency.*

**Supplement Table 1**

Comparison of Demographic Characteristics Between the Two Groups Before and After Screening

| **Variables** | **Category** | Before  363(100%) | After  356(100%) | $t/X^{2}$ | *P* |
| --- | --- | --- | --- | --- | --- |
| Age | y≤65 | 85(23.42%) | 81(22.75%) | 0.060 | 0.970 |
|  | 65＜y≤75 | 218(60.06%) | 217(60.96%) |  |  |
|  | y＞75 | 60(16.53%) | 58(16.29%) |  |  |
| Sex | Male | 154(42.42%) | 150(42.13%) | 0.006 | 0.938 |
|  | Female | 209(57.58%) | 206(57.87%) |  |  |
| Marital Status | Currently single | 79(21.76%) | 78(21.91%) | 0.002 | 0.961 |
|  | Married with partner | 284(78.24%) | 278(78.09%) |  |  |
| CCI Score | 0 | 208(57.30%) | 205(57.58%) | 0.006 | 0.997 |
|  | 1 | 119(32.78%) | 116(32.58%) |  |  |
|  | ≥2 | 36(9.92%) | 35(9.83%) |  |  |
| Anesthesia Type | Spinal anesthesia | 226(62.26%) | 221(62.08%) | 0.003 | 0.957 |
|  | General anesthesia | 137(37.74%) | 135(37.92%) |  |  |
| Prior Knee Arthroplasty | No | 294(80.99%) | 288(80.90%) | 0.002 | 0.964 |
|  | Yes | 69(19.01%) | 68(19.10%) |  |  |
| Patients | Mainland | 147(40.50%) | 143(40.17%) | 0.007 | 0.932 |
|  | Hong Kong | 216(59.50%) | 213(59.83%) |  |  |
| Hospital Stay Duration | | 9.34±3.49 | 9.31±3.50 | 0.115 | 0.908 |
| Preoperative days | | 3.12±1.82 | 3.10±1.83 | 0.131 | 0.896 |
| Postoperative days | | 6.22±2.93 | 6.21±2.94 | 0.042 | 0.966 |
| Medical service fees | | 10568.02±2008.62 | 10461.56±2031.07 | 0.703 | 0.482 |
| Examination/laboratory fees | | 2504.92±890.15 | 2538.03±813.63 | -0.548 | 0.584 |
| Material costs | | 13128.19±1228.53 | 12833.82±1152.11 | 2.867 | 0.004** |
| Medication costs | | 736.31±430.67 | 724.04±420.04 | 0.388 | 0.698 |
| hospital-acquired infections | | 0 | 0 | - | - |
| Total costs | | 26937.45±3182.62 | 26557.44±2985.97 | 1.516 | 0.130 |

**Supplement Table 2**

Mediation Effect Test of the Impact of Preoperative Hospitalization Days on Hospitalization Costs by Whether the Patient is from Hong Kong

| Variable | Mediator: Preoperative days | | | | Dependent Variable: Hospitalization Costs | | | |  |
| --- | --- | --- | --- | --- | --- | --- | --- | --- | --- |
|  | *Coeff* | *Boot Mean* | *LLCI* | *ULCI* | *Coeff* | *Boot Mean* | *LLCI* | *ULCI* |  |
| constant | 2.81 | 2.83 | 1.92 | 3.72 | 23468.58 | 23492.52 | 21893.77 | 25058.50 |  |
| HK patient | -0.11 | -0.11 | -0.50 | 0.26 | -302.14 | -330.37 | -1584.41 | 846.51 |  |
| Married with partner | -0.34 | -0.35 | -0.95 | 0.20 | -797.51 | -783.03 | -1501.86 | -48.98 |  |
| General anesthesia | 0.05 | 0.05 | -0.36 | 0.49 | 981.56 | 978.40 | 385.01 | 1605.02 |  |
| Prior knee arthroplasty | -0.37 | -0.38 | -0.82 | 0.09 | -841.76 | -841.80 | -1531.87 | -136.77 |  |
| Female | 0.28 | 0.28 | -0.03 | 0.62 | 130.65 | 135.09 | -423.17 | 697.18 |  |
| Age (ref: ≤65 years) | | | | | | | | |  |
| 65＜y≤75 | 0.16 | 0.16 | -0.24 | 0.54 | 231.94 | 229.18 | -400.90 | 806.83 |  |
| y＞75 | 0.50 | 0.50 | -0.11 | 1.21 | 1295.37 | 1309.59 | 315.58 | 2380.99 |  |
| CCI score (ref: 0) | | | | | | | | |  |
| 1 | -0.11 | -0.11 | -0.51 | 0.30 | 1079.67 | 1070.59 | 428.08 | 1729.93 |  |
| ≥2 | 0.29 | 0.28 | -0.54 | 1.13 | 1394.48 | 1380.38 | 342.91 | 2484.16 |  |
| Preoperative days |  |  |  |  | 568.33 | 555.92 | 282.57 | 820.57 |  |
| *R^2^*=0.184，*P*=0.214 | | | | | *R^2^*=0.510，*P*＜0.001 | | | |  |
|  | *Effect* | *BootSE* | *BootLLCI* | *BootULCI* |  |  |  |  |  |
| X→Y | -534.45 | 295.10 | -1114.88 | 45.98 |  |  |  |  |  |
| X→M→Y | -52.82 | 98.54 | -268.26 | 133.90 |  |  |  |  |  |

*The analysis excluded variables with VIF >2 to mitigate multicollinearity.*

*Bootstrap samples = 5000, Confidence level for confidence intervals = 95%.*

The mediation effect of ‌preoperative hospitalization days‌ was ‌not statistically significant‌.

**Supplemental Table 3**‌
Mediation Effect Test of Postoperative Hospitalization Days on Hospitalization Costs by Whether the Patient is from Hong Kong‌

| Variable | Mediator: Preoperative days | | | | Dependent Variable: Hospitalization Costs | | | |  |
| --- | --- | --- | --- | --- | --- | --- | --- | --- | --- |
|  | *Coeff* | *Boot Mean* | *LLCI* | *ULCI* | *Coeff* | *Boot Mean* | *LLCI* | *ULCI* |  |
| constant | 6.11 | 6.11 | 4.33 | 7.90 | 20892.66 | 20899.81 | 19734.45 | 22082.52 |  |
| HK patient | -0.83 | -0.83 | -1.48 | -0.21 | -20.23 | -27.59 | -464.11 | 397.86 |  |
| Married with partner | -0.89 | -0.90 | -1.85 | -0.02 | -358.78 | -371.10 | -968.51 | 177.46 |  |
| General anesthesia | 0.45 | 0.44 | -0.16 | 1.06 | 700.85 | 705.14 | 256.70 | 1170.39 |  |
| Prior knee arthroplasty | -0.11 | -0.10 | -0.85 | 0.71 | -965.02 | -964.48 | -1519.12 | -397.76 |  |
| Female | 0.08 | 0.08 | -0.49 | 0.67 | 240.23 | 245.39 | -143.60 | 640.30 |  |
| Age (ref: ≤65 years) | | | | | | | | |  |
| 65＜y≤75 | 0.19 | 0.20 | -0.47 | 0.87 | 194.67 | 196.89 | -318.52 | 664.02 |  |
| y＞75 | 1.23 | 1.22 | 0.24 | 2.30 | 707.43 | 700.73 | -9.53 | 1397.47 |  |
| CCI score (ref: 0) | | | | | | | | |  |
| 1 | 0.64 | 0.64 | -0.08 | 1.37 | 581.41 | 574.02 | 102.67 | 1057.50 |  |
| ≥2 | 0.43 | 0.44 | -0.48 | 1.41 | 1272.51 | 1260.58 | 438.93 | 2077.56 |  |
| Postoperative days |  |  |  |  | 680.31 | 680.00 | 606.51 | 762.22 |  |
| *R^2^*=0.266，*P* =0.002 | | | | | *R^2^*=0.759，*P*＜0.001 | | | |  |
|  | *Effect* | *BootSE* | *BootLLCI* | *BootULCI* |  |  |  |  |  |
| X→Y | -20.23 | 224.58 | -461.95 | 421.49 |  |  |  |  |  |
| X→M→Y | -562.71 | 216.34 | -992.24 | -142.46 |  |  |  |  |  |

*The analysis excluded variables with VIF >2 to mitigate multicollinearity.*

*Bootstrap samples = 5000, Confidence level for confidence intervals = 95%.*

Postoperative hospitalization days‌ ‌fully mediated‌ the effect of whether the patient is from Hong Kong on hospitalization costs.‌

**Supplemental Table 4**‌
Mediation Effect Test of Medical Service Costs on Hospitalization Costs by Whether the Patient is from Hong Kong

| Variable | Mediator: Medical service fees | | | | Dependent Variable: Hospitalization Costs | | | |  |
| --- | --- | --- | --- | --- | --- | --- | --- | --- | --- |
|  | *Coeff* | *Boot Mean* | *LLCI* | *ULCI* | *Coeff* | *Boot Mean* | *LLCI* | *ULCI* |  |
| constant | 9465.42 | 9460.41 | 8290.50 | 10678.30 | 14026.74 | 14054.52 | 12397.34 | 15585.88 |  |
| HK patient | -350.85 | -350.49 | -789.79 | 63.18 | -1192.01 | -1172.98 | -3085.67 | 701.06 |  |
| Married with partner | -557.17 | -559.89 | -1191.66 | 30.93 | -304.42 | -304.73 | -647.88 | 31.13 |  |
| General anesthesia | 611.91 | 613.73 | 189.38 | 1047.07 | 288.29 | 289.84 | -57.73 | 652.61 |  |
| Prior knee arthroplasty | -347.75 | -346.03 | -844.88 | 156.43 | -635.34 | -641.34 | -1078.44 | -216.12 |  |
| Female | 168.82 | 171.65 | -229.29 | 565.44 | 106.52 | 103.81 | -219.85 | 427.75 |  |
| Age (ref: ≤65 years) | | | | | | | | |  |
| 65＜y≤75 | 302.26 | 303.75 | -145.26 | 756.01 | -33.67 | -34.78 | -455.36 | 340.54 |  |
| y＞75 | 1119.32 | 1122.06 | 463.89 | 1815.06 | 167.66 | 166.95 | -437.24 | 750.52 |  |
| CCI score (ref: 0) | | | | | | | | |  |
| 1 | 466.81 | 470.24 | -17.74 | 967.76 | 428.54 | 423.12 | 89.19 | 760.54 |  |
| ≥2 | 553.14 | 543.42 | -110.91 | 1225.52 | 895.72 | 885.69 | 304.70 | 1476.35 |  |
| Medical service fees |  |  |  |  | 1.17 | 1.16 | 1.03 | 1.30 |  |
| *R^2^*=0.318，*P* <0.001 | | | | | *R^2^*=0.876，*P*＜0.001 | | | |  |
|  | *Effect* | *BootSE* | *BootLLCI* | *BootULCI* |  |  |  |  |  |
| X→Y | -134.71 | 166.79 | -462.77 | 193.36 |  |  |  |  |  |
| X→M→Y | -443.89 | 275.24 | -994.39 | 80.95 |  |  |  |  |  |

*The analysis excluded variables with VIF >2 to mitigate multicollinearity.*

*Bootstrap samples = 5000, Confidence level for confidence intervals = 95%.*

Medical service costs‌ ‌partially mediated‌ the effect of whether the patient is from Hong Kong on hospitalization costs.

**Supplemental Table 5**‌
Mediation Effect Test of Diagnostic and Testing Costs on Hospitalization Costs by Whether the Patient is from Hong Kong‌

| Variable | Mediator: Examination/laboratory fees | | | | Dependent Variable: Hospitalization Costs | | | |  |
| --- | --- | --- | --- | --- | --- | --- | --- | --- | --- |
|  | *Coeff* | *Boot Mean* | *LLCI* | *ULCI* | *Coeff* | *Boot Mean* | *LLCI* | *ULCI* |  |
| constant | 2404.46 | 2404.89 | 1985.39 | 2835.06 | 19992.95 | 20016.97 | 17658.03 | 22346.00 |  |
| HK patient | -191.61 | -190.81 | -351.34 | -33.81 | -126.45 | -150.99 | -2124.29 | 1848.57 |  |
| Married with partner | -164.68 | -166.27 | -373.83 | 38.90 | -620.00 | -620.50 | -1351.33 | 81.80 |  |
| General anesthesia | 115.48 | 116.71 | -44.40 | 276.25 | 766.38 | 755.78 | 234.29 | 1295.91 |  |
| Prior knee arthroplasty | -402.73 | -403.42 | -590.71 | -213.54 | -199.85 | -201.77 | -840.22 | 405.70 |  |
| Female | -19.07 | -17.60 | -175.78 | 142.54 | 332.61 | 325.31 | -172.51 | 836.84 |  |
| Age (ref: ≤65 years) | | | | | | | | |  |
| 65＜y≤75 | 82.70 | 80.08 | -76.03 | 233.74 | 152.26 | 160.42 | -404.38 | 701.89 |  |
| y＞75 | 572.01 | 568.34 | 296.30 | 854.57 | 350.15 | 360.59 | -564.91 | 1247.88 |  |
| CCI score (ref: 0) | | | | | | | | |  |
| 1 | 342.20 | 342.12 | 190.50 | 500.79 | 302.63 | 305.64 | -292.63 | 892.77 |  |
| ≥2 | 709.88 | 707.93 | 302.22 | 1192.55 | 80.05 | 80.53 | -857.94 | 1006.87 |  |
| Examination/laboratory fees |  |  |  |  | 2.10 | 2.10 | 1.44 | 2.81 |  |
| *R^2^*=0.468，*P* <0.001 | | | | | *R^2^*=0.641，*P*＜0.001 | | | |  |
|  | *Effect* | *BootSE* | *BootLLCI* | *BootULCI* |  |  |  |  |  |
| X→Y | -184.49 | 265.83 | -707.35 | 338.36 |  |  |  |  |  |
| X→M→Y | -389.39 | 178.29 | -774.25 | -69.08 |  |  |  |  |  |

*The analysis excluded variables with VIF >2 to mitigate multicollinearity.*

*Bootstrap samples = 5000, Confidence level for confidence intervals = 95%.*

Diagnostic and testing costs‌ ‌fully mediated‌ the effect of whether the patient is from Hong Kong on hospitalization costs.

**Supplemental Table 6**‌
Mediation Effect Test of Consumable Costs on Hospitalization Costs by Whether the Patient is from Hong Kong

| Variable | Mediator: Material costs | | | | Dependent Variable: Hospitalization Costs | | | |  |
| --- | --- | --- | --- | --- | --- | --- | --- | --- | --- |
|  | *Coeff* | *Boot Mean* | *LLCI* | *ULCI* | *Coeff* | *Boot Mean* | *LLCI* | *ULCI* |  |
| constant | 12740.85 | 12747.27 | 11987.45 | 13531.05 | 12355.36 | 12572.29 | 6901.08 | 19038.83 |  |
| HK patient | 70.52 | 68.08 | -190.11 | 332.99 | -1522.26 | -1642.20 | -8962.47 | 4868.70 |  |
| Married with partner | -164.01 | -164.51 | -466.49 | 118.35 | -791.46 | -791.18 | -1584.00 | -58.01 |  |
| General anesthesia | 135.95 | 137.78 | -115.60 | 396.94 | 876.56 | 874.46 | 334.23 | 1449.50 |  |
| Prior knee arthroplasty | -262.03 | -260.89 | -642.87 | 91.59 | -769.81 | -768.96 | -1425.50 | -119.59 |  |
| Female | 56.96 | 53.41 | -211.60 | 310.86 | 229.52 | 219.31 | -284.68 | 719.68 |  |
| Age (ref: ≤65 years) | | | | | | | | |  |
| 65＜y≤75 | -76.49 | -76.53 | -345.28 | 193.65 | 406.90 | 406.20 | -157.72 | 972.08 |  |
| y＞75 | -224.41 | -223.67 | -673.65 | 210.93 | 1776.04 | 1776.52 | 887.89 | 2716.44 |  |
| CCI score (ref: 0) | | | | | | | | |  |
| 1 | 52.29 | 49.15 | -222.92 | 325.70 | 957.04 | 959.88 | 353.95 | 1592.20 |  |
| ≥2 | 183.04 | 179.26 | -283.56 | 591.68 | 1363.29 | 1349.72 | 306.74 | 2454.70 |  |
| Material costs |  |  |  |  | 1.00 | 0.98 | 0.49 | 1.39 |  |
| *R^2^*=0.148，*P* =0.561 | | | | | *R^2^*=0.560，*P*＜0.001 | | | |  |
|  | *Effect* | *BootSE* | *BootLLCI* | *BootULCI* |  |  |  |  |  |
| X→Y | -658.27 | 283.97 | -1216.80 | -99.74 |  |  |  |  |  |
| X→M→Y | 74.99 | 140.85 | -199.67 | 371.90 |  |  |  |  |  |

*The analysis excluded variables with VIF >2 to mitigate multicollinearity.*

*Bootstrap samples = 5000, Confidence level for confidence intervals = 95%.*

The mediation effect of ‌consumable costs‌ was ‌not statistically significant‌.

‌**Supplemental Table 7**‌
Mediation Effect Test of Medication Costs on Hospitalization Costs by Whether the Patient is from Hong Kong

| Variable | Mediator: Medication costs | | | | Dependent Variable: Hospitalization Costs | | | |  |
| --- | --- | --- | --- | --- | --- | --- | --- | --- | --- |
|  | *Coeff* | *Boot Mean* | *LLCI* | *ULCI* | *Coeff* | *Boot Mean* | *LLCI* | *ULCI* |  |
| constant | 437.64 | 439.48 | 228.17 | 633.42 | 23732.60 | 23391.02 | 21367.65 | 25143.98 |  |
| HK patient | -111.00 | -110.55 | -199.99 | -31.21 | -1534.88 | -1117.28 | -2578.42 | 850.45 |  |
| Married with partner | -81.13 | -80.10 | -177.75 | 19.13 | -610.89 | -558.04 | -1241.42 | 79.21 |  |
| General anesthesia | 145.04 | 144.42 | 46.03 | 265.17 | 497.46 | 462.84 | -34.19 | 956.55 |  |
| Prior knee arthroplasty | -26.85 | -26.99 | -109.93 | 58.38 | -916.40 | -915.81 | -1497.58 | -309.87 |  |
| Female | 86.94 | 85.85 | 9.68 | 180.01 | 7.12 | -13.80 | -494.84 | 460.51 |  |
| Age (ref: ≤65 years) | | | | | | | | |  |
| 65＜y≤75 | 16.10 | 15.38 | -149.32 | 132.99 | 176.14 | 124.82 | -430.08 | 668.87 |  |
| y＞75 | 75.80 | 75.22 | -95.76 | 214.65 | 1128.87 | 1055.29 | 232.93 | 1936.05 |  |
| CCI score (ref: 0) | | | | | | | | |  |
| 1 | 155.34 | 155.99 | 60.69 | 240.02 | 237.08 | 193.35 | -408.19 | 800.73 |  |
| ≥2 | 118.42 | 119.08 | -12.52 | 249.48 | 959.35 | 911.19 | 10.45 | 1859.98 |  |
| Medication costs |  |  |  |  | 3.34 | 3.93 | 2.39 | 6.52 |  |
| *R^2^*=0.334，*P*＜0.001 | | | | | *R^2^*=0.665，*P*＜0.001 | | | |  |
|  | *Effect* | *BootSE* | *BootLLCI* | *BootULCI* |  |  |  |  |  |
| X→Y | 25.29 | 264.26 | -494.49 | 545.07 |  |  |  |  |  |
| X→M→Y | -589.95 | 230.21 | -1091.69 | -169.46 |  |  |  |  |  |

*The analysis excluded variables with VIF >2 to mitigate multicollinearity.*

*Bootstrap samples = 5000, Confidence level for confidence intervals = 95%.*

Medication costs‌ ‌fully mediated‌ the effect of whether the patient is from Hong Kong on hospitalization costs.
